# 3D carbon crystals: theoretical prediction and experimental preparation

**DOI:** 10.1093/nsr/nwaf125

**Published:** 2025-03-29

**Authors:** Yanbo Zhang, Fei Pan, Kun Ni, Yanwu Zhu

**Affiliations:** Hefei National Research Center for Physical Sciences at the Microscale, University of Science and Technology of China, Hefei 230026, China; Department of Materials Science and Engineering, School of Chemistry and Materials Science, University of Science and Technology of China, Hefei 230026, China; Hefei National Research Center for Physical Sciences at the Microscale, University of Science and Technology of China, Hefei 230026, China; Department of Materials Science and Engineering, School of Chemistry and Materials Science, University of Science and Technology of China, Hefei 230026, China; Hefei National Research Center for Physical Sciences at the Microscale, University of Science and Technology of China, Hefei 230026, China; Department of Materials Science and Engineering, School of Chemistry and Materials Science, University of Science and Technology of China, Hefei 230026, China; Hefei National Research Center for Physical Sciences at the Microscale, University of Science and Technology of China, Hefei 230026, China; Department of Materials Science and Engineering, School of Chemistry and Materials Science, University of Science and Technology of China, Hefei 230026, China; State Key Laboratory of Precision and Intelligent Chemistry, University of Science and Technology of China, Hefei 230026, China

**Keywords:** 3D carbon crystal, structure prediction, experimental preparation, charge injection

## Abstract

*Sp, sp* ^2^ or *sp*^3^ hybridization of carbon atoms results in a linear, triangular or tetrahedral configuration of bonding geometry, respectively. By combining different hybridizations in one structure, a variety of 3D carbon allotropes with periodic crystal structures can be obtained with potential novel properties and applications. With the rapid development of computational capability in recent years, a large number of new 3D carbon structures have been proposed with their properties predicted; the development of new experimental techniques has also led to the successful experimental preparation of several carbon crystals. Observing the rapid advancement of 3D carbons subsequent to the breakthroughs in 2D graphene, this paper reviews the recent progress in constructing carbon crystals by summarizing the structural design and specifically highlighting the preparation using template carbonization, organic synthesis, high-pressure processing and charge injection.

## Teaser

This review summarizes the recent progress in constructing 3D carbon crystals, including theoretical predictions and experimental preparation through template carbonization, organic synthesis, high-pressure processing and charge injection.

## INTRODUCTION

Designing carbon structures with desired properties has been the focus in the field of carbon research for a long time. Connecting carbon atoms with different hybridizations leads to an essentially countless number of carbon allotropes, providing opportunities to tune the properties of carbons, as the hybridization type largely determines the physical and chemical features of the formed structures. In recent decades, the extensive investigation of fullerenes [1], carbon nanotubes (CNTs) [2], graphene [3] and other low-dimensional carbon materials [4] has verified the crucial role of structural regulation. For example, superconductivity is found in doped C_60_ and magic-angle twisted graphene [5,6]. Flat bands and electronic phase separation are identified in multilayer rhombohedral graphene [7,8], which is distinct from the extensively studied hexagonal phase of graphene stacking. Whether the single-walled carbon nanotube (SWCNT) is metallic or semiconductive depends on the chirality as well [9]. To utilize nanocarbons in practical scenarios, methods such as DNA-mediated assembly of CNT arrays [10], cross-linking gelation of graphene oxide [11], 3D printing of graphene superstructures [12], and template-directed growth of graphene networks [13] have been developed to assemble carbon nanostructures into bulk materials. Through such non-covalent assembly or reconstruction of nanomaterials by violent chemical reaction, the macroscopic carbons generally own random structures or fragile properties [14]. Designing 3D carbons from nanostructures while maintaining certain periodicity can not only give full play to the structural flexibility, but also bring about new insights for carbon materials science.

The construction of 3D carbon crystals, other than the two most stable structures, graphite and diamond, is challenging. From the point of view of thermodynamics, all carbons tend to transform to *sp*^2^-hybridized graphite at very high temperatures/ambient pressure or to *sp*^3^-hybridized diamond at high pressure/high temperature (HPHT) conditions. Since many 3D carbon crystals are thermodynamically metastable and can hardly be obtained by simple phase transition or carbonization, very careful control of parameters is necessary for the preparation. Recent successful synthesis of graphene nanoribbons and CNTs from polycyclic aromatic hydrocarbons (PAHs) has demonstrated the feasibility of bottom-up fabrication from carbon units [15,16], which may be extended to the preparation of 3D carbon structures. Theoretical calculations have proposed the possibility of building pillared graphene or carbon foam [17,18], via the connection of CNTs or graphene, respectively. Introducing the negative Gaussian curvature, Mackay and Terrones in 1991 proposed ‘schwarzite’ (named after German mathematician Hermann Schwarz, who originally defined the triply periodic minimal surfaces) by topologically extending *sp*^2^ carbon into a periodic 3D space [19]. Simulations indicate that these periodic negatively curved carbons containing six- and eight-membered rings possess massless Dirac fermions [20], and their electronic properties may be regulated from metallic to semiconducting [21,22], showing potential applications in gas adsorption, ion storage, catalysis and so on [23–27].

Several routes have been explored for the preparation of 3D carbon crystals, starting from carbon clusters, molecules or nanostructures. By controlling the pyrolysis and deposition of hydrocarbon precursors in periodic microporous zeolite templates, zeolite-templated carbons (ZTCs) with structures replicating those of templates were prepared [28]. Through bottom-up organic synthesis, some carbon fragments with negative curvatures such as octabenzo[8]circulene [29] and monkey saddle-shaped nanographene [30] have been synthesized, which can serve as building blocks for carbon schwarzites, yet their covalent assembly into an atomically periodic 3D structure is still on the way. By treating carbon nanostructures such as graphene, CNTs and fullerenes at HPHT conditions, covalent bonds form between nanocarbons accompanied by further structural phase transitions, resulting in the experimental preparation of several superhard carbon phases such as M- [31], Z- [32] and V-carbons [33].

Clearly, studies on 3D carbon crystals are still in the very early stages. Published theoretical prediction results have included the electronic structure of thermodynamically stable phases [34], while the possible reaction path starting from the precursors remains elusive. In the limited number of reports on the experimental preparation of periodic 3D crystals, the microstructures often contain a large number of defects [35–37]. Furthermore, the amount of sample is restricted when some preparations are performed at extreme conditions [33,38]. On the other hand, the crystals of Mg_2_C_60_ [39,40] and Mg_4_C_60_ [40,41] have been prepared, in which C_60_ molecules are covalently connected with each other in the plane but non-covalently stacked out of plane. Hou *et al.* [40] and Meirzadeh *et al.* [41] utilized organic cation slicing and mechanical exfoliation to further exfoliate the Mg_4_C_60_ crystals into single/few layers of 2D C_60_ polymer, respectively, after removing Mg from the crystals. The preliminary characterizations based on these 2D polymers show that they have enhanced thermal and electric conductivity as a result of the in-plane covalent bonding, compared with molecular C_60_. In addition, our group highlighted the important role of interface charge injection to explain the phase transition of graphite from rhombohedral (3R) to hexagonal (2H) [42], and the connection of C_60_ cages to polymer crystals and long-range ordered porous carbon (LOPC) crystals [43]. The charge injection method may offer an effective approach for constructing new carbons much like Lego blocks, through the precise control of interfaces between nanocarbons.

With the purpose of providing researchers in this field with a comprehensive understanding of state-of-the-art 3D carbon crystal research, here we summarize the relevant progress, including theoretical predictions of some typical structures and experimental preparation techniques of template carbonization, organic synthesis, high pressure processing and so on. The charge-injection-assisted synthesis will be highlighted in more detail with regard to its potential in terms of cost effectiveness and feasibility in practical applications.

## THEORETICAL PREDICTIONS

The versatile bonding of carbon atoms provides fertile ground for the discovery and design of new carbon materials. The theoretical prediction of new carbon allotropes can be traced back to the 1960s. In 1966, David H. Jones imagined that if carbon atoms are arranged in a pattern of pentagons and hexagons, they could be connected to each other in a low-density spheroidal form [44]. C_60_ was experimentally discovered in 1985 [1]. Since then, extensive efforts have been devoted to predicting the structure and properties of new carbon crystals based on first-principles calculations. To predict new structures, algorithms including *ab initio* random structure searching (AIRSS) [45], particle swarm optimization (PSO) [46], stochastic surface walking (SSW) [47], universal structure predictor: evolutionary xtallography (USPEX) [48], genetic algorithm (GA) [49] and the multi-objective inverse band structure design method [50], have been utilized. On the other hand, pressure can be applied to transform a structure into a new one, by which various superhard but lightweight carbon structures can be constructed by altering the arrangement of stacked CNTs and the compression angle and position [51]. In typical theoretical predictions, three steps are taken [52]: (i) Random structures are generated from selected elements and structures. (ii) After relaxation, candidates are screened by various criteria such as energy, phonon spectrum, and thermal and mechanical stability, in which energy is the prime factor. (iii) Properties of these predicted carbons are obtained by calculating the electronic structure at the ground state.

Theoretical prediction has made significant contributions in the field of carbon research by offering a framework with possible stable structures along with interesting chemical and physical properties. In recent decades, with the improvement in computational methods, more carbon crystal structures have been proposed, such as graphene-based all *sp*^2^ hybridized [53] and graphdiyne-based *sp*-*sp*^2^ mixed hybridization carbons [54], 3D *sp*^2^ graphitic carbon represented by carbon schwarzites [21], *sp*^3^ diamond-like structure [55], 3D *sp*^2^-*sp*^3^ mixed hybridization carbon foams [18], and *sp*-*sp*^3^ mixed yne-diamond structure [56]. As of February 2024, a total of 1661 3D carbon allotropes have been predicted using density functional theory (DFT) and are listed in the Samara Carbon Allotrope Database (SACADA) [57]. In the following we show some of them by classifying the structures in terms of hybridization of carbon atoms.

### 3D *sp*^3^ carbon crystals

**Figure 1.**
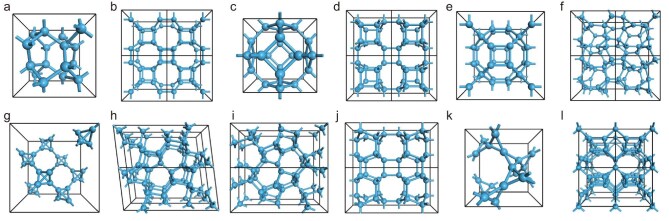
Crystal structures of representative 3D *sp*^3^ carbons. (a) BC-8. (b) Supercubane-C8. (c) Bcc-C6. (d) Bct-C4. (e) FCC-C32. (f) Sc-C20. (g) T-carbon. (h) M-carbon. (i) W-carbon. (j) Z-carbon. (k) K6-carbon. (l) T-C9.

In theoretical studies, most 3D *sp*^3^ carbon structures were proposed to explain or predict new carbon crystals prepared at high pressures, which are usually superhard, insulating and transparent. Figure 1 summarizes some typical structures.

**BC-8** is an ultra-dense *sp*^3^ diamond-like structure (density: 3.52 g/cm^3^, comparable to 3.5 g/cm^3^ of diamond) proposed in 1980s [58,59] to explain experimentally obtained ultra-dense crystals. BC-8 is expected to be transformed from diamond at pressures above 1 TPa. BC-8 has a body-centered cubic (bcc) structure and a space group of *Ia*-3 with a lattice constant of 4.49 Å. Despite research efforts with regard to high-pressure processed carbons [60,61], the experimental evidence on BC-8 is still lacking. In 2023, Shi *et al.* used molecular dynamic (MD) simulations based on machine-learning potential to predict a thermodynamic pathway for obtaining BC-8 from diamond in a double-shock compression experiment [62]. A similar pathway was also predicted by Nguyen-Cong *et al.* in 2024, in which diamond is compressed to a supercooled liquid for the subsequent nucleation and growth of BC-8 [63]. **Supercubane-C8** was proposed by Strelnitskii *et al.* to explain their experimental discovery of a super-dense cubic carbon [64], which consists of 3D interconnected cubane. Initially, they thought that supercubane-C8 belongs to the *Im*-3 space group with a lattice constant of 4.28 Å and density of 4.1 g/cm^3^. In 1989, Hoffmann *et al.* argued that the C-C bond in the proposed supercubane-C8 structure is too short to be reasonable and pointed out that the space group of supercubane-C8 should be *Im*-3*m* [59]. Instead, Hoffmann *et al.* suggested that BC-8 can better explain the experiment of Strelnitskii *et al.*  **Bcc-C6** is a cubic carbon with Sodalite structure, first reported by Beagley *et al.* in 1992 [65]. Its space group is *Im*-3*m* with a lattice constant of 4.392 Å and a density of 2.826 g/cm^3^. In 2006, Cohen *et al.* investigated the electronic properties and structural stability of Bcc-C6 with calculations, and demonstrated that it is a semiconductor with an indirect bandgap of 2.5 eV and a bulk modulus of ∼350 GPa [66]. In 2012, using DFT and electron diffraction calculations, Pokropivny *et al.* [67] reported that Bcc-C6 is more suitable than BC-8 or Supercubane-C8 to explain the ultra-dense cubic carbon crystals prepared by Strelnitskii *et al.*, by considering the lower total energy of Bcc-C6 and the better match between the diffraction patterns and experimental results.

**Bct-C4** can be regarded as square C_4_ rings connected to each other, and the space group is *I*4/*mmm*. In 1997, Baughman *et al.* performed molecular mechanics simulations to investigate the structure and properties of Bct-C4. Based on the studies of structure, formation heat, bulk modulus and diffraction patterns, they proposed a ‘rectangulated diamond’ to explain the experimental findings of quasi lonsdaleite, such as the instability at ambient conditions associated with graphitization [68]. Bct-C4 was also proposed by Domingos *et al.* in 2004 by simulating the transition of CNT bundles under compression [69]. Bct-C4 can also be theoretically constructed by the phase transition of compressed graphite, which was used by Umenoto *et al.* [70] to explain the superhard carbon prepared by cold compressing graphite. **FCC-C32** has a space group of *Fm*-3*m* [71], a lattice constant of 6.62 Å and a density of 2.75 g/cm^3^. Each cell contains 32 carbon atoms, of which 8 *sp*^3^ carbon atoms are located on the body diagonal, connecting four adjacent cubane structures. Found by He *et al.* using a particle swarm optimization method [72], **Sc-C20** belongs to the *P*2_1_3 space group with a lattice constant of 4.886 Å, containing 20 carbon atoms in each cell. Sc-C20 is kinetically stable with high mechanical strength (bulk modulus of 403.29 GPa, shear modulus of 416.82 GPa) and wide bandgap (4.20 eV). It is very likely to be one of the experimentally reported i-carbons with a lattice constant of *a* = 0.486 nm, typically produced at ambient pressures by ion-beam deposition [73] and radiofrequency plasma decomposition of hydrocarbon gases [74].

**T-carbon** is constructed by replacing each carbon atom in diamond with a carbon tetrahedron, proposed by Sheng *et al.* using first-principles calculations [55]. The angle between C−C bonds in the tetrahedron is 60°, and the bond angle between the adjacent tetrahedral carbons is 144.74°. The calculations of structure, electronic properties and phonon spectra indicate that T-carbon is a stable semiconductor with a density of 1.50 g/cm^3^ and direct bandgap of ∼3.0 eV. Because of the existence of cavities inside this structure, the Vickers hardness is estimated to be ∼61 GPa, 33% weaker than diamond. **M-carbon** was proposed by Oganov *et al.* using first-principles calculations combined with evolutionary algorithms [75], and has been used to explain the superhard transparent carbon prepared by cold compressing graphite [76]. M-carbon has a monoclinic space group *C*2/*m* with lattice constants of *a* = 9.089 Å, *b* = 2.496 Å and *c* = 4.104 Å, in which each cell contains 16 carbon atoms. The calculations show that M-carbon is more stable than graphite at high pressure and could be obtained by specific covalent bonding between adjacent graphite layers. The pressure required for the graphite to M-carbon phase transition was predicted to be ∼13.4 GPa [76], close to that for the formation of cold-compressed graphite (∼14 GPa) [77]. M-carbon is a transparent, superhard material with a calculated hardness of 83 GPa and bandgap of 3.6 eV.

**W-carbon** is another superhard candidate obtained by cold-compressing graphite [78], belonging to orthorhombic space group *Pnma*, consisting of alternating seven- and five-membered rings. The lattice constants are *a* = 8.979 Å, *b* = 2.496 Å and *c* = 4.113 Å, and each cell contains 16 carbon atoms. W-carbon is thermodynamically more stable than M-carbon, with a higher bulk modulus of 444.5 GPa and thus requires a lower phase transition pressure of 13.32 GPa. **Z-carbon**, also known as Cco-C8, was proposed by Zhao *et al.* in 2011 [79] to explain the superhard transparent carbon experimentally obtained by compressing CNTs. Z-carbon belongs to the orthorhombic space group *Cmmm*, with lattice parameters of *a* = 8.674 Å, *b* = 4.209 Å and *c* = 2.487 Å, and 16 carbon atoms in each cell. Calculations by Zhao *et al.* have shown that Z-carbon is more stable and has a greater Vickers hardness and bulk modulus than W-carbon or M-carbon. In addition, Z-carbon can be theoretically obtained via structural phase transition of graphite, consisting of alternating four- and eight-membered rings. Amsler *et al.* [80] used it to explain the material obtained by cold compressing graphite as well.

**K6-carbon** is a cubic carbon structure predicted by Niu *et al.* in 2014 [81], and has six atoms in a primitive cell comprising *sp*^3^-hybridized C_3_ triangle rings and a space group of *I*4_1_32 (*O*^8^). The calculated phonon dispersion and elastic constant indicate that K6-carbon is a stable, highly ductile structure with a density lower than graphite. The band structure calculations show that it is a metallic carbon with an electronic density of states of ∼0.10 states/eV per atom at the Fermi level, which is very different from other reported wide-bandgap *sp*^3^ carbons. **T-C9** is a superhard *sp*^3^-hybridized tetragonal carbon structure with a space group of *P*-4*m*2 proposed by Liu *et al.* in 2020 [82]. T-C9 is mechanically and dynamically more stable than T-carbon at ambient pressure. The calculated bulk modulus, shear modulus and hardness are 328, 243 and 66.7 GPa, respectively. T-C9 has a wide indirect bandgap of 4.128 eV and may find potential application in electronic and optoelectronic devices.

### 3D *sp*^2^ carbon crystals

**Figure 2.**
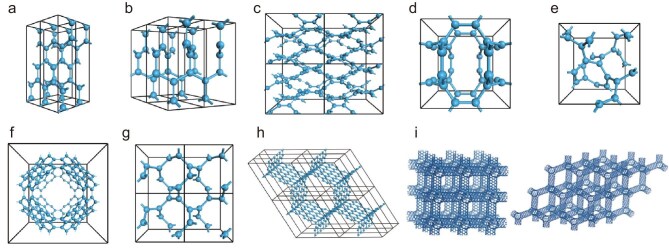
Crystal structures of representative 3D *sp*^2^ carbons. (a) Bct-4. (b) H-6. (c) HS-C48. (d) CP-C20. (e) CP-C24. (f) P-192 carbon schwarzite. (g) K4-carbon. (h) Triangular carbon foam. (i) 3D orthogonal and diamond-like CNT networks. Adapted with permission from ref. [98]. Copyright 2011 American Chemical Society.

Figure 2 shows several 3D *sp*^2^ carbon structures. Among them, **Bct-4**, proposed by Hofmann *et al.* [83] in 1983, is regarded as a pioneering work on non-graphitic *sp*^2^ carbon crystals. The authors referred to the crystal structure of thorium disilicide (ThSi_2_) for constructing Bct-4, which is metallic and belongs to the *I*4_1_/*amd* space group with lattice constants of *a* = *b* = 2.52 Å and *c* = 8.57 Å, and a density of 2.94 g/cm^3^. **H-6** was proposed by Tamor and Hass in 1990 [84]. First-principles simulations show that H-6 is metallic, belonging to the *P*6_2_22 space group with optimized lattice constants of *a* = *b* = 2.645 Å and *c* = 6.375 Å, containing six equivalent carbon atoms in each cell. **HS-C_48_** is a *sp*^2^-hybridized 3D carbon with a honeycomb structure and 48 carbon atoms in a unit cell (density: 2.277 g/cm^3^) [85], which belongs to the *Pbam* space group with lattice constants of *a* = 7.734 Å, *b* = 6.594 Å and *c* = 7.514 Å. Calculations indicate that it is a metallic carbon with massless Dirac fermions. **CP-C20** is a *sp*^2^-hybridized hollow carbon network [86], belonging to the *Pm*-3 space group with 20 atoms in a unit cell. The phonon spectrum and *ab initio* MD calculations confirm its stability. **CP-C24** is another stable porous metallic *sp*^2^ carbon with a symmetry of *P*4_1_32 and cubic cell of 24 carbon atoms [87]. The calculations show that it is stable up to 1500 K. Three peaks of the calculated X-ray diffraction (XRD) of cP-C24 are matched with experimental results obtained from detonation soot [88]. With a stable and porous 3D conductive network structure, cP-C24 may find potential applications in molecule adsorption or energy storage.

Carbon schwarzite was proposed by Mackay and Terrones in 1991 [19] by periodically extending *sp*^2^ carbon atoms into a 3D space with negative Gaussian curvatures, which can be divided into D-, P- and G-type according to the different topological features. A typical structure belonging to the *Im*-3*m* space group, **P-192**, was given in the initial paper. Vanderbilt and Tersoff used coalescent C_60_ molecules to construct D-168 [89], which belongs to the *Fd*3 space group. Lenosky *et al.* proposed D-216 and P-216 and calculated their electrical and mechanical properties [21,90]. By adopting the three-coordinate srs topological structure as a carbon structure, **K4-carbon** was reported by Itoh *et al.* in 2009 [91]. First-principles calculations reveal that K4-carbon exhibits a metallic property originating from the twisted *π* state above the Fermi surface. To investigate the stability of K4-carbon, Yao *et al.* [92] analyzed its phonon-band structure and Liang *et al.* [93] calculated its elastic constants. They both proved that K4-carbon is unstable at ambient conditions. However, the mechanical stability results obtained from the phonon spectrum are sensitive to computational accuracy, as a low numerical accuracy may result in the generation of imaginary frequency modes [94].

Theoretical calculations have also proposed a route to 3D carbons by connecting graphene or CNT fragments with *sp*^2^ bonds. For example, zigzag graphene nanoribbons are connected to form honeycombed 3D *sp*^2^ networks with variable pore sizes [95]. In 2011, a type of triangular carbon foam was designed, in which graphene nanoribbons are joined together by carbon hexagons at the joints [96], as shown in Fig. 2h. *Ab initio* calculations indicate that this **triangular carbon foam** is metallic with a mass density of 1.67 g/cm^3^ and a bulk modulus of 202 GPa. The structure is energetically favorable, indicated by its cohesive energy being lower than that of graphite. Similarly, by constructing SWCNT junctions with negative curvature via the introduction of seven- and five-membered ring pairs, nanotubes with different chirality can be covalently interconnected to make 3D CNT super-architectures [97]. As shown in Fig. 2i, Zeng *et al.* proposed several 3D CNT networks, including **orthogonal** and **diamond-like network** structures. *Ab initio* calculations suggest that most of these CNT networks are metallic, regardless of the electronic properties of the original CNT units [98]. The same idea can be used to construct 3D periodic carbon schwarzites from low-dimensional structural motifs such as fullerenes, CNTs and graphene.

### Mixed hybridization carbon crystals

**Figure 3.**
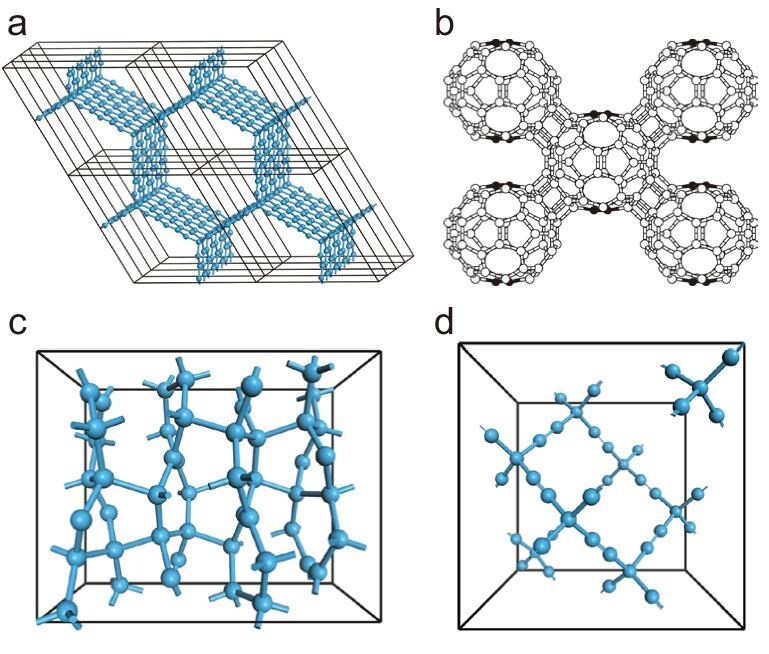
Structures of several mixed hybridization carbons. (a) Karfunkel's carbon. (b) 3D C_60_ polymer (tri-coordinated atoms are shown in black). Adapted with permission from ref. [100]. Copyright 2000 American Physical Society. (c) oC48. (d) Yne-diamond.

*Sp* ^2^-*sp*^3^ mixed hybridization 3D carbons can be obtained by organizing 2D *sp*^2^ networks with a certain number of *sp*^3^ bonds between layers. In 1992, Karfunkel *et al.* constructed several periodic *sp*^2^-*sp*^3^ carbon networks by connecting graphene nanoribbons at the edges with *sp*^3^ bonds [18], and a typical structure of **Karfunkel's carbon** is shown in Fig. 3a. First-principles calculations show that these structures are thermodynamically stable with metallic properties. In 2001, Tomanek *et al.* proposed a series of mixed hybridization carbon foams based on diamond and graphite by introducing *sp*^3^ hybridization connections between graphite fragments [99]. DFT calculations indicate that these carbon foams are rigid and metallic with porosity and low density, and can be potentially utilized as molecular sieves and hydrogen storage materials. In 2000, Burgos *et al.* explored possible superhard carbon phases by simulating the 3D covalent connection of C_60_ using DFT methods and semiempirical potentials [100]. Two body-centered orthorhombic phases and one bcc phase were proposed, having 52, 56 and 60 *sp*^3^-hybridized carbon atoms per C_60_ molecule, respectively, among which the one with 56 tetracoordinated carbons is shown in Fig. 3b. These **3D C_60_ polymers** are semiconducting, with a shear modulus of ∼240 GPa and a bulk modulus of ∼300 GPa. **oC48** is another 3D carbon crystal consisting of *sp*^2^ and *sp*^3^ bonding, in which each cell has 48 carbon atoms, as shown in Fig. 3c. DFT calculations [101] indicate that there are four types of bonding length (1.4782 Å, 1.6551 Å, 1.5790 Å and 1.3336 Å) in oC48, and the optimized lattice constants are *a *= 9.4076 Å, *b* = 4.3435 Å and *c *= 7.3597 Å. oC48 is predicted to be a superhard semiconductor material with an indirect bandgap of 1.464 eV and a hardness of 73.3 GPa. **Yne-diamond** [102], as shown in Fig. 3d, also known as super-diamond, is a diamond-like 3D carbon crystal composed of *sp*^3^- and *sp*-hybridized carbon atoms, and can be constructed by attaching an acetylene unit to every two carbon atoms in the diamond. The *sp*^3^-*sp* and *sp*-*sp* bonds in yne-diamond are much stronger than the *sp*^3^-*sp*^3^ bonds in diamond [103]. Itzhaki *et al.* therefore predicted it to be a superhard material, particularly when the voids are filled with Xe atoms [104]. Bu *et al.* [56] performed first-principles calculations to further study the mechanical properties and structural stability of yne-diamond in 2012 and found it a metastable carbon structure with a density of 0.926 g/cm^3^, about one-quarter of that of diamond. The calculated shear and Young's modulus were 14.8 and 19.3 GPa, respectively, only 2.7% and 1.8% of those of diamond, demonstrating that yne-diamond is not a superhard material.

From the very limited examples above we can see that *sp*^3^-hybridized carbon crystals generally are superhard and wide bandgap insulators, which may be useful in the fields of grinding, machining, semiconductors and aerospace. Many models have been proposed to explain the superhard transparent carbons obtained from high pressure experiments, in which T-carbon has been synthesized by laser irradiation of CNTs [105], and M-carbon by the cold compression of graphite at 15-20 GPa [106]. BC-8 has been predicted but not yet obtained by double-shock compression [62]. *Sp*^2^-hybridized carbon crystals are generally metallic and flexible, and many are porous and expected to be used in adsorption, ion sieving and energy storage. Among them, carbon foam was synthesized in 2016 by deposition of vacuum-sublimated graphite [107], and cP-24 cP-C24 by detonation experiment [87]. By combining *sp, sp*^2^ and *sp*^3^ hybridization, the mixed hybridization carbon crystals may have excellent mechanical properties and adjustable electronic properties. Several 3D C_60_ polymers have been synthesized by HPHT [38,108,109], and an interpenetrating graphene network has been synthesized by compressing glassy carbon [110].

For many structures not listed here, readers can refer to recent review articles [34,111–113] and the SACADA database. At present, most of the theoretical studies have analyzed the stability of new carbon structures with calculations of mechanical and electronic properties, but very little has been done to suggest possible preparation pathways. In the following we will review some typical experimental strategies for preparing 3D carbon crystals.

## EXPERIMENTAL PREPARATION OF 3D CARBON CRYSTALS

Strictly speaking, only a very small portion of theoretically predicted 3D carbon crystals have been prepared in the laboratory and the research on their property characterizations is even rarer. In past years, the raw materials for the preparation of 3D carbon crystals mainly included graphite and amorphous carbon, especially in HPHT preparations. With the rapid advancement of the mass production of carbon nanomaterials such as fullerenes, CNTs and graphene, the development of new strategies may be expected based on such carbon nanomaterials. In the following we briefly summarize the typical experimental techniques of 3D carbon crystals, and would like to invite readers to notice the difference between theoretical models and experimental results.

### Template-assisted preparation

Template-assisted preparation has been used to prepare carbon materials with reasonable structure control. To make crystals, carbon precursors are introduced into the periodic pores of the templates by chemical vapor deposition (CVD) or monomer-impregnation-polymerization for subsequent carbonization in the confined space. The templates are etched away and ordered porous carbons with an external structure identical to the internal surface of templates are obtained. Here we summarize two typical template carbons prepared in the experiment which are very close to the 3D crystalline microporous carbons, i.e. ZTCs and ordered carbonaceous frameworks (OCFs), synthesized by the hard template method and self-template method, respectively.

#### Zeolite-templated carbons

Zeolites are a class of aluminosilicate-based microporous crystals. The International Zeolite Association has recorded over 200 types of zeolites [114] with distinct pore structures, many of which have interconnected nanochannels similar to the internal space of schwarzites. For instance, the channel structure of FAU zeolite closely resembles the internal space of D-type schwarzite. Owing to their great thermal stability, adjustable catalytic activity and diversity of pore structure, zeolites have long been considered ideal templates to prepare microporous carbon crystals. ZTCs can be seen as the negative replicas of their parent templates.

**Figure 4.**
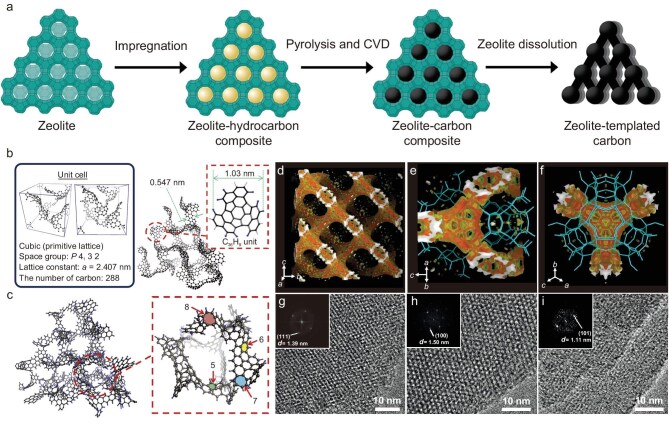
Preparation of ZTCs via the hard template method. (a) Schematic diagram of the typical zeolite template method. Adapted with permission from ref. [120]. Copyright 2020 American Chemical Society. (b) An open graphene framework model for ZTC [Model-I]. (c) A long-range ordered 3D graphene geometric structure model with more structural heterogeneity for ZTC [Model-II]. Adapted with permission from ref. [118]. Copyright 2018 Elsevier. (d–f) 3D electron-density map of the carbon framework formed after carbon deposition in zeolite FAU at 600°C. (g–i) TEM images and (insets) Fourier diffractions of template-free carbon, generated using a template of La^3+^-ion-exchanged (g) FAU, (h) EMT or (i) beta zeolites. Adapted with permission from ref. [28]. Copyright 2016 Springer Nature.

The first attempt to synthesize 3D ordered carbons from zeolites was reported by Kyotani *et al.* in 1997 [115]. The authors proposed two synthetic routes, including CVD and impregnation-carbonization, to introduce carbon source into the internal channels of zeolite FAU-Y and control the carbonization. After releasing the zeolite template, the obtained carbon was named ZTC. Afterwards, they improved the synthesis using a two-step carbon filling method (Fig. 4a) and reported a 3D periodic microporous carbon material with a specific surface area of 4100 m^2^/g [35,116], whose structure was explained by an open graphene-like framework model (Model-I, Fig. 4b) constructed from buckybowl-like structural motifs (C_36_H_9_) containing carbon hexagons and pentagons [117]. In 2018, the same group proposed a more realistic structural model (Model-II, Fig. 4c) by introducing more structural heterogeneity and using curved graphene fragments containing six-, seven- and eight-membered rings as structural motifs [118]. This model can be described as a 3D interconnected carbon framework consisting of diverse open-blade-type carbon moieties with partially formed closed-strut moieties, an intermediate between Model-I and ideal schwarzite with a tubular structure. The above-mentioned ZTCs are typically prepared through the decomposition of carbon sources at temperatures above 600°C. However, the high temperature processing may reduce the selectivity of reactions, resulting in challenges such as the over-deposition of carbon on the external surface or in the internal channels of zeolite, preventing the inward diffusion and reaction of carbon sources [119].

A metric that is useful in determining the templating fidelity of ZTCs is structural packing density (SPD_cell_), which has been defined elsewhere as the mass of carbonaceous material inside the channels divided by the mass of the zeolite template [120]. Schwarzite-like ZTCs would necessarily exhibit a high SPD_cell_ of 0.63–0.71 whereas the reported experimental ZTCs typically exhibit an SPD_cell_ of 0.32–0.40 [121]. Several methods have been developed to improve the deposition of carbon inside pores, e.g. using pulsed CVD to alter the deposition kinetics [122] or using a nano-sized zeolite template to shorten the diffusion distance of carbon sources [123]. Up to now, the highest SPD_cell_ values obtained by the pulsed CVD method and using nano-sized zeolites are 0.53 and 0.68, respectively [121]. In these cases, over-deposition often happens, resulting in the formation of core-shell-like ZTCs with graphitic shells on the outer surface.

To further increase the SPD_cell_ of ZTCs, the catalytic activity of the pore walls must be precisely controlled to optimize the adsorption and decomposition selectivity of carbon precursors. Zeolites can undergo a substitution of tetravalent silicon with trivalent aluminum (Al^3+^), requiring the introduction of an extra-framework charge-balancing cation, which can be exchanged with a wide range of cations, such as H^+^, Na^+^, K^+^, Ca^2+^, NH_4_^+^, La^3+^ and Y^3+^. Previous studies have investigated the role of different cations in modulating the catalytic activity of zeolites and the synthesis of ZTCs [124]. A breakthrough was reported in 2016 [28], when Ryoo *et al.* achieved the preparation of microporous 3D graphene-like ZTCs by introducing lanthanide metal cations (La^3+^, Y^3+^) into pores of zeolites via the solution-based ion-exchange method (Fig. 4d–i), in which La^3+^ functions as a catalyst to reduce the carbonization temperature of ethylene or acetylene. The low reaction temperature prevents the deposition of carbon on the external surface of the template, thus enabling the selective formation of a 3D graphene-like framework within the zeolite channels. They proposed a model constructed from structural motifs containing 258 carbon atoms to describe the experimentally obtained ZTC and indicated that it may be a type of carbon schwarzite. Similarly, a variety of zeolites with different shapes and pores (FAU, EMT, beta, LTL, MFI, LTA) can be used to prepare high-quality microporous carbon nanostructures. In addition, by introducing Ca^2+^ into X zeolite instead of La^3+^, another ZTC with 3D graphene structure and nanotube-like framework along the inner surface of zeolite could be prepared via carbonization of ethylene, providing a high yield, significantly reduced cost and tremendous potential for large-scale production and practical application [125].

To find out whether the experimentally synthesized ZTCs can be regarded as real schwarzite and to better describe the atomic structure of existing ZTCs, in 2018 Braun *et al.* [126] developed a computational method based on the Monte Carlo algorithm to simulate the synthesis process of ZTCs, which can generate the corresponding ZTC structure from parent zeolites. They also established a library of ZTC models that show promise for experimental synthesis and proposed criteria for selecting zeolites suitable for preparing ZTCs. It has been proposed that ZTC is an incarnate of schwarzite. However, Stadie *et al.* [120] argued that none of the zeolite templates proposed by Braun *et al.* [126] could be used to prepare carbon schwarzite, for three reasons: (i) Carbon schwarzite is composed of a pair of interpenetrating networks with the same surface area and volume. (ii) There should not be too many edge states such as H- and O-containing functional groups on carbon schwarzite. (iii) The reported SPD_cell_ value is too low to prove that a closed 3D structure consists of fullerene-like nodes connected by tubular struts. Thus, the ZTC structures presented by Braun *et al.* [126] can be regarded as ‘unbalanced schwarzites’ [120]. Recently, Chi *et al.* demonstrated that the atomic structure of ZTCs is sensitive to the synthesis conditions [127]. Comprehensive experimental characterizations and theoretical calculations indicated that the ZTCs prepared by carbon deposition using large molecule precursors and subsequent framework densification at low temperatures are mainly composed of open-blade-type moieties, exhibiting low surface curvature and abundant H-terminated edge sites. On the other hand, small molecule precursors and high densification temperature generate ZTCs with an increased portion of closed-strut carbon moieties, featuring large surface curvature and diminished edge site.

#### Ordered carbonaceous frameworks

Another method for constructing 3D crystalline microporous carbons is the self-template method, which mainly involves direct pyrolytic carbonization of crystalline organic frameworks. Over the past decade, pyrolytic carbons based on metal-organic frameworks [128] and covalent organic frameworks [129,130] have been widely investigated, but the key is how to retain the ordered structure of the precursors after carbonization.

**Figure 5.**
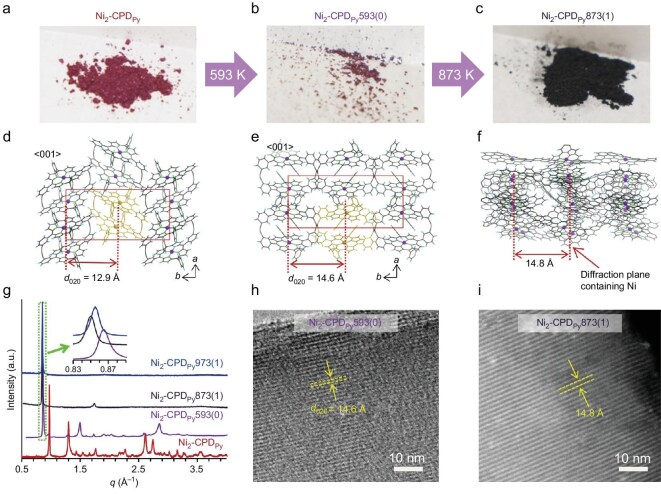
Preparation of ordered carbonaceous frameworks via the self-template method. (a–c) Structural transformation and optical photographs of Ni_2_-CPD_Py_*X*(*Y*) under heat treatment, where *X* and *Y* represent the heat treatment temperature (K) and the holding time (h), respectively. (d–f) Molecular-level structures of (d) pristine Ni_2_-CPD_Py_, (e) Ni_2_-CPD_Py_593(0) and (f) Ni_2_-CPD_Py_873(1). (g) XRD patterns of Ni_2_-CPD_Py_ heat-treated at different temperatures. (h, i) TEM images of (h) Ni_2_-CPD_Py_593(0) and (i) Ni_2_-CPD_Py_873(1). Adapted with permission from ref. [36]. Copyright 2017 Springer Nature.

To prepare molecular-scale ordered 3D carbon crystal, Nishihara *et al.* [36] in 2017 designed a synthetic strategy, which includes first strengthening the crystal framework by solid-state polymerization of crystalline precursor molecules and then carbonizing the polymer while maintaining its original structure. The schematic of synthesis and crystal structures is shown in Fig. 5. For the preparation, nickel-containing cyclic porphyrin dimer (Ni_2_-CPD_Py_), a molecular crystal with a thermally stable nickel-porphyrin center and polymerizable diacetylene moieties, is used as the precursor. Upon heating to 593 K at an inert atmosphere, polymerization between diacetylene moieties occurs, by which the precursor molecules are transformed into a crystalline polymer and then carbonized into a highly conductive ordered carbon framework at above 873 K. The carbon obtained is essentially a 3D ordered framework assembled from non-stacked graphene sheets, and the porphyrin Ni-N_4_ units in the precursor are retained and regularly distributed in the graphene sheets, forming a clear (020) plane as observed in transmission electron microscopy (TEM). The successful preparation and structural regulation of ordered carbonaceous frameworks make it possible to manipulate the structure of 3D carbon crystals at the molecular scale.

Despite reports, we have to say that atomically periodic 3D carbon crystals have not been verified through a template-assisted method. The obtained interconnected graphene frameworks often have an open-blade-type structure, low carbon packing density and substantial amounts of H and O. One possible factor in the preparation of ZTCs is related to the incomplete carbon source filling, channel blockage and external surface deposition. By precisely controlling the growth process to exactly mimic the structure at a molecular level, ZTCs and OCFs may be further optimized to prepare new 3D carbon crystals.

### Assembly of negatively curved polycyclic aromatic hydrocarbons

In order to accurately synthesize carbon schwarzites and other negatively curved 3D carbon crystals, a bottom-up organic synthesis strategy is to prepare PAH segments with negative curvature, which are then used as templates or building blocks to construct macroscopic 3D carbon structures. This method has been applied to the preparation of low-dimensional carbon structures. For example, high quality nanoporous graphene [131] and graphene nanoribbons [15] have been fabricated by depositing planar PAH precursors onto metal substrates followed by heat treatment. Single chiral CNTs have been selectively produced by cyclodehydrogenation and epitaxial growth using designed PAHs as templates [16].

**Figure 6.**
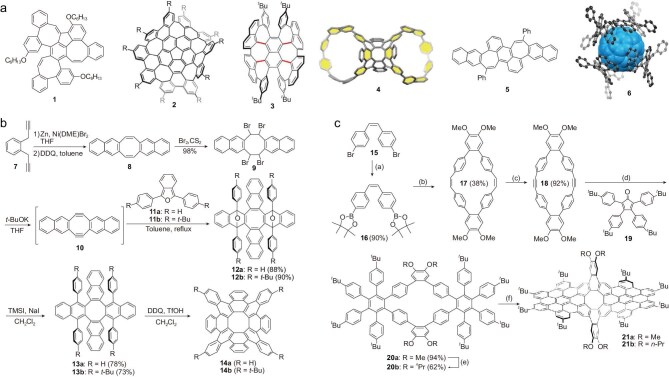
Synthesis of negatively curved molecular carbons. (a) Several negatively curved PAHs containing seven- or eight-membered rings. Adapted with permission from refs [30,137,139–142]. Copyright 2019 John Wiley and Sons; copyright 2013 Springer Nature; copyright 2023 Elsevier; copyright 2023 American Chemical Society; copyright 2019 John Wiley and Sons; copyright 2022 American Chemical Society. (b, c) Typical synthetic routes for preparing PAHs containing [8]circulene units by (b) in-out and (c) out-in cyclization. Adapted with permission from refs [29,138]. Copyright 2019 American Chemical Society; copyright 2017 John Wiley and Sons.

The key to the bottom-up synthesis of carbon schwarzites lies in the precise design of nanographene precursors with negative curvature. These negatively curved graphene segments are units that carry structural information about carbon schwarzites and can be synthesized by introducing seven- and eight-membered rings into PAH molecules. The main idea is to build the larger polycyclic aromatic systems by adding varying numbers of benzene rings to [7]circulene or [8]circulene. It is generally believed that the study of negatively curved PAHs began with the synthesis of [7]circulene by Yamamoto *et al.* in 1983 [132]. Since then, the design, synthesis and properties of negatively curved conjugated carbon nanostructures containing five-, seven- and eight-membered rings have garnered much attention, and remarkable progress has been made in recent decades. Several representative structures are shown in Fig. 6a.

In 2013, Wu *et al.* synthesized two kinds of peri-substituted [8]circulenes through a 4-fold Pd-catalyzed annulation of tetraiodotetraphenylene with diarylethynes [133]. For the preparation of tetrabenzo[8]circulene, Suzuki and Sakamoto developed an out-in cyclization approach in 2013 [134]. First, a macrocyclic molecule was synthesized by a Suzuki-Miyaura cross-coupling reaction of o-dibromobenzene and o-terphenylboronic ester, then an eight-membered ring was formed through intramolecular connection of the macrocyclic compound by Scholl reaction. Later in 2014, Whalley *et al.* reported an in-out cyclization strategy [135], by employing the double Diels-Alder reaction of sulfoxide with Sondheimer-Wong diyne to produce tetraphenylene derivatives and subsequent intramolecular Pd-catalyzed arylation to form more benzene rings around the eight-membered rings. Two years later, Whalley and co-workers improved the synthesis route by Scholl reaction of tetraphenylidene derivatives, resulting in the production of functionalized tetrabenzo[8]circulene with a higher yield [136]. The construction of these [8]circulene derivatives provides the possibility of synthesizing structural units of carbon schwarzites if suitable precursors are selected, promoting the investigation of negatively curved molecular carbons.

Many negatively curved PAHs have been designed and experimentally synthesized based on out-in cyclization and in-out cyclization strategies. In 2013, Itami *et al.* synthesized a grossly warped C_80_H_30_ with 26 rings including heptagons and pentagons derived from a corannulene [137]. In 2019, Miao *et al.* successfully synthesized octabenzo[8]circulene [29], as shown in Fig. 6b. In the preparation, precursor molecules like Sondheimer-Wong diyne were firstly prepared by 2-fold Diels-Alder reactions with 1,3-diarylisobenzofurans, and then deoxidation and aromatization of intermediate species were achieved by reduction using iodotrimethylsilane and sodium iodide. The final octabenzo[8]circulenes were obtained through Scholl reaction after adding fluoromethanesulfonic acid (TfOH) and 2,3-dichloro-5,6-dicyano-1,4-benzoquinone (DDQ). Following an out-in cyclization strategy similar to Suzuki's approach, they also synthesized two types of twisted nanographene in 2017 by Scholl reaction [138]. The specific synthetic route is shown in Fig. 6c. Firstly, macrocyclic diene was prepared by a Suzuki-Miyaura cross-coupling reaction of boronic acid ester with 1,2-dibromo-4,5-dimethoxybenzene, then transformed into macrocyclic diyne after bromination and subsequent elimination of hydrogen bromide. After a 2-fold Diels-Alder reaction between macrocyclic diyne and cyclopentadienone, and after removing carbonyl and undergoing subsequent Scholl reactions under strongly acidic conditions (TfOH) with DDQ as the oxidant, twisted nanographene was finally obtained with 14 intramolecular C-C bonds formed inside. In 2020, Mastalerz *et al.* synthesized another negatively curved PAH containing three eight-membered rings [30] by starting from the hexyloxy-substituted truxene. An intermediate compound was obtained by bromination and Suzuki-Miyaura cross-coupling reaction, and then three eight-membered rings were formed by alkali-catalyzed condensation and cyclization after adding potassium tert-butoxide. In 2023, Miao *et al*. synthesized several negatively curved molecular nanocarbons containing multiple heptagons by Scholl reaction of macrocyclic precursors [139]. By attaching C-shaped paraphenylene precursors to 2,11,18,27-tetrabromooctabenzo[8]circulene with subsequent intramolecular Yamamoto coupling and reductive aromatization reactions, Miao *et al.* synthesized two new molecular nanocarbons combining both negatively curved and tubular structures [140]. In 2019, Würthner *et al.* reported the synthesis of a highly warped, *sp*^2^ carbon scaffold containing heptagons in only two steps from unfunctionalized alkene precursors [141], which can be seen as a substructure of a previously predicted carbon schwarzite. In 2022, the same group synthesized a fullerene-embedded schwarzite fragment, which is composed of fullerenes embedded in four equivalent negatively curved PAHs [142]. In 2021, Miao *et al.* reported the synthesis of a 3D porous covalent network through the polymerization of the tetrabromo derivative of octabenzo[8]circulene by the nickel-mediated Yamamoto coupling reaction, which has a specific surface area of 732 m^2^/g and excellent lithium storage performance [143].

It is worth noting that these molecular carbons can not only act as precursors to be assembled into 3D carbons but also serve as prototypes to investigate structure–property relationships. Based on the success in organic synthesis of graphene nanoribbons and CNTs using PAHs, it is not difficult to imagine that if appropriate catalysts and supporting templates are found, through polymerization, on-surface reaction or stacking into covalent organic frameworks, a variety of carbon nanomaterials with negative curvature can be assembled in 3D space to achieve more accurate synthesis of 3D carbon crystals like schwarzites.

### High-pressure processing

As an important physical parameter, the pressure can effectively regulate the interatomic/intermolecular interaction and bonding, and thus the structure of carbons [144–148]. The most common observation is that graphite transforms into diamond at HPHT. Based on covalent connection, polymerization or phase transitions of carbon building blocks such as graphene, CNTs and fullerenes, new 3D carbon crystals can be synthesized under high-pressure conditions.

#### Cold compression of graphite

Graphite is typically composed of graphene layers stacked in the sequence of ABAB…, which is prone to interlayer slipping and *sp*^2^-*sp*^3^ phase transition at HPHT, and generally used to produce high-quality cubic diamond in industries. However, the situation is completely different at high pressure and room temperature or high pressure with shear stress applied, where graphite may transform into *sp*^3^-hybridized superhard carbon crystals including M-carbon, hexagonal diamond and so on.

Since the 1960s, numerous experiments have identified the occurrence of structural phase transition when graphite is compressed to 14–17 GPa at room temperature. Electrical, optical, XRD and Raman spectroscopy characterizations indicate that graphite slowly transforms into a *sp*^3^-hybridized, transparent and insulating phase, commonly known as cold compressed graphite [149–152]. Different from the high-pressure transformation into diamond at high temperature or with large shear stress applied, this phase transition is reversible and relaxed samples will revert to graphite after decompression [153]. Recently, an *in-situ* Raman spectroscopy on compression of highly oriented pyrolytic graphite (HOPG) showed that the *sp*^2^-*sp*^3^ phase transition starts at 9.7 GPa [154], while another work on compression of carbon nanowalls reported a phase transition at 5.9 GPa [155].

**Figure 7.**
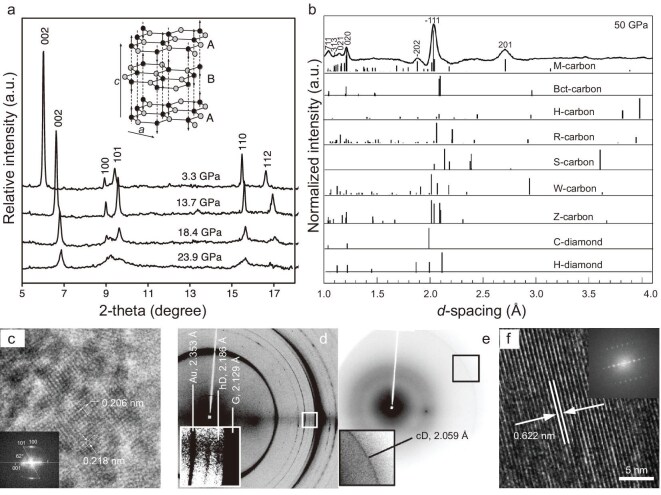
Preparation of superhard, transparent carbon crystals by cold compressing graphite. (a) Hydrostatic XRD patterns for graphite compressed at ambient temperature. The inset image shows the phase transition from graphite to M-carbon. Adapted with permission from ref. [31]. Copyright 2003 American Association for the Advancement of Science. (b) XRD pattern obtained after compressing graphite to ∼50 GPa compared with the calculated results of several new carbon structures, in addition to cubic and hexagonal diamond. Adapted with permission from ref. [158]. Copyright 2012 Springer Nature. (c) High-resolution TEM and corresponding fast Fourier transform (inset) images of lonsdaleite. Adapted with permission from ref. [162]. Copyright 2016 Inderscience Enterprises Ltd. (d, e) XRD patterns of compressed and sheared graphite (d) at 0.4 GPa with 45° anvil rotation and (e) after quenching to room pressure from 0.7 GPa. (f) High-resolution TEM and fast Fourier transforms (inset) of an orthorhombic structure. Adapted with permission from ref. [163]. Copyright 2019 Elsevier.

In 2003 Mao *et al.* synthesized a superhard transparent carbon crystal with hardness close to diamond by cold compressing graphite at ∼16.7 GPa [31]. *In-situ* inelastic X-ray scattering suggests that the interlayer graphitic *π* bonding is reduced by 50%, and it was believed that graphite transforms into a distorted monoclinic phase or orthorhombic structure at this pressure. To explain the mechanical and optical properties and XRD (Fig. 7a) results of this superhard carbon, theoretical work based on *ab initio* structural search algorithms proposed a variety of structural model candidates [70,76,78–80,156,157], including M-, S-, R-, W-, Z-, H and Bct-carbon, all containing four-, five-, seven- or eight-membered rings in addition to hexagonal carbon rings. Later in 2012, Wang *et al.* made a detailed study of the structural transition of HOPG under high pressure [158], and found that M-carbon was the most probable structure by comparing the XRD data obtained from experiments with several structures predicted theoretically (Fig. 7b). Although the experimental Raman spectra are in good agreement with the calculated M-, Z- and Bct-carbons, M-carbon is the most recognized structure of the superhard phase of cold-compressed graphite at present. It is generally believed that graphite gradually transforms into M-carbon above 20 GPa, and the M-carbon phase remains stable up to >50 GPa. However, Schindler *et al.* found some cubic and hexagonal diamonds in the released products of graphite after it was compressed up to 70 GPa by Raman spectroscopy, suggesting that M-carbon may further transform into diamond at sufficiently high pressures [159].

The introduction of shear stress may promote structural phase transition under high pressures, resulting in the formation of new 3D carbon crystals from graphite at room temperature, but the depressurized samples may revert into diamond [160]. Aksenenkov *et al.* found that applying a rotational shear to M-carbon at a higher pressure leads to a further structural phase transition, and hexagonal and cubic diamonds are obtained after decompression from 17 GPa and 19–25 GPa, respectively [161]. Blank *et al.* also observed similar results, i.e. that hexagonal graphite transforms into rhombohedral graphite mixed with hexagonal and cubic diamond after decompression [162]. The TEM image of experimentally obtained hexagonal diamond crystal is shown in Fig. 7c. Gao *et al.* reported that diamond could be prepared at room temperature and below 1 GPa by applying sufficiently strong shear stress [163]. After repeating compression and shear treatment of graphite, they obtained hexagonal and cubic diamonds decompressed from 0.4 and 0.7 GPa, respectively, as shown in Fig. 7d and e. Such pressures are significantly lower than that required for the graphite-diamond phase transition in the conventional phase diagram. In addition, an unknown orthorhombic phase was found in the sample decompressed from 3 GPa (Fig. 7f), which may be the previously predicted orthorhombic carbon phase Cco-C_8_, also known as Z-carbon [79]. The experimental results show that applying high pressure combined with strong shear (such as ball milling) may be an effective strategy to synthesize new 3D carbon crystals.

Shock compression has been used to mimic the shock of meteorites since microscopic diamonds were found in remnants of explosively driven graphite in 1961 [164]. In 1967, lonsdaleite, i.e. hexagonal diamond, which is theoretically harder than cubic diamond [165], was identified inside the fragments of the Canyon Diablo meteorite [166]. In 2003, Yamada *et al.* prepared a new simple cubic carbon with a lattice parameter of 0.514 nm by shock compressing a mixture of carbon black and tetracyanoethylene [167]. A bcc carbon structure, BC12 [168], was theoretically proposed to explain its structure, while another group [169] argued that Sc-C20 is closer to the experimental results of Yamada *et al.* In 2016, Kraus *et al.* found the formation of lonsdaleite above 170 GPa from pyrolytic graphite [170], while another *in-situ* XRD study in 2017 indicated that shock-compressed HOPG is transformed into lonsdaleite at 50 GPa [171]. On the other hand, dynamic shock compression driven by explosives [172], gas guns [173] or pulsed high-energy lasers [174] can also simulate high-pressure phase transition. Due to the small sample size and short timescales (nanoseconds to microseconds), it is challenging to detect phase transitions in laboratory shock experiments [175], leading to debate about the dynamic process of the shock preparation of diamond and lonsdaleite [176], as well as conclusions about meteor impacts [177].

#### Cold compression of carbon nanotubes

CNTs can be regarded as rolled graphene and may be transformed into new 3D carbon crystals at high pressure on account of their tubular structure. Early theoretical studies [178–180] predicted the possibility of polymerization of SWCNTs under high pressure, and the later experimental TEM observations on the electron beam-induced coalescence of CNT bundles [181], welding of SWCNTs [182] and covalent bonding of nanotubes in bundles [183] support this view.

**Figure 8.**
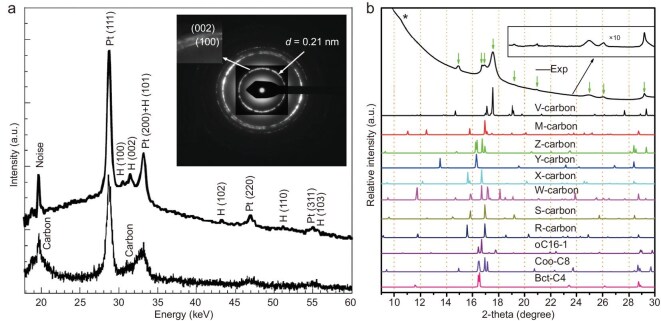
Cold compression of carbon nanotubes (CNTs). (a) XRD patterns of (bottom) starting phase of CNT bundles and (top) recovered phase released from 100 GPa. The inset image shows the electron diffraction pattern of the recovered sample. Adapted with permission from ref. [32]. Copyright 2004 National Academy of Sciences. (b) XRD patterns of experimentally obtained V-carbon by cold compressing C_70_ peapods compared with the simulated result and previously proposed post-graphite phases. Adapted with permission from ref. [33]. Copyright 2017 American Physical Society.

In the past few decades, experimental studies have been conducted on the high-pressure transition of CNTs at room temperature. Raman spectroscopic, electrical and XRD studies [184–187] show that both single-walled and multi-walled CNTs undergo reversible *sp*^2^-*sp*^3^ phase transition near the pressure for reversible conversion of graphite to M-carbon, indicating that the high-pressure phases of CNTs and graphite are closely related. In 2004, Wang *et al.* found that when a pressure of ∼75 GPa is applied, CNTs transform into a dense and superhard phase, which can be retained after decompression [32]. Raman spectroscopic and XRD (Fig. 8a) studies indicate that it adopts a hexagonal *sp*^3^ structure, which differs from hexagonal diamond, while its bulk modulus approaches that of diamond. Zhai *et al.* [188] indicated that the structure of this new phase is consistent with the theoretically predicted Z-carbon.

On the other hand, CNTs are less likely to transform into new structures under high pressures compared with graphite, possibly due to the inertness of the tube-like structure and the disordered arrangement between tubes. To overcome the problem of low reactivity, fullerene molecules have been embedded into CNTs to construct a peapod structure as the precursor. In 2017, Yang *et al.* synthesized a superhard carbon by compressing C_70_ peapods to a pressure of higher than 60 GPa at room temperature, and named it V-carbon [33]. Detailed XRD, Raman, electron diffraction, scanning transmission electron microscopy and energy dispersive X-ray spectroscopic characterizations prove that this all-*sp*^3^ monoclinic carbon is composed of five-, six- and seven-membered rings, and maintains stability at ambient conditions. Theoretical calculations indicate that the hardness and bulk modulus are 89 GPa and 411 GPa, respectively. Figure 8b shows the comparison of experimentally obtained XRD patterns with several theoretically predicted carbon structures, proving the successful synthesis of V-carbon. Although numerous thermodynamically stable carbon structures derived from CNTs have been theoretically proposed, few of them have been experimentally prepared. Selecting tightly packed CNT bundles or ordered networks with specific arrangement, intersection and stacking as precursors, new 3D carbon crystals may be obtained by combining the roles of high pressure, high temperature and catalysts.

#### Compression of fullerenes

Fullerenes are electron-deficient polyenes with a high degree of freedom and a high molecular symmetry, which can form rich covalent bonds with surrounding molecules through cycloaddition reactions, therefore forming various polymers. By regulating the polymerization or coalescence of fullerenes, it is expected to synthesize a series of theoretically predicted 3D carbon crystals.

The compression of fullerenes was carried out in early 1990s to determine the bulk modulus and phase transition of C_60_ molecular crystal, since theoretical studies predicted that C_60_ is stiffer than diamond [189–191]. The bulk modulus of face-centered cubic (fcc) phase C_60_ molecular crystal at atmospheric-pressure and room temperature was reported to be ∼10 GPa, a typical characteristic of isotropic van der Waals intermolecular bonding [192]. The volume compressibility −d(ln*V*)/d*P* is 7.0 ± 1 × 10^−12^ cm^2^/dyne, 3 and 40 times the values for graphite and diamond, respectively [193]. The rapid, non-hydrostatic compression of C_60_ to pressures of 20 ± 5 GPa at room temperature would transform it instantaneously into bulk polycrystalline diamond [194]. On the other hand, high pressure can shorten the molecular distance and change the orientation of fullerenes, which is conducive to the formation of intermolecular covalent bonds. In 1994, Iwasa *et al.* first reported that the polymerization of C_60_ could be achieved by applying a pressure of 5 GPa at moderate temperature [195]. Theoretical analysis indicated that this process involves a [2 + 2] cycloaddition reaction between two 6–6 double bonds of adjacent C_60_ molecules to form cyclobutene rings and formation of 1D chains by sequentially connecting C_60_ [196]. Since then, a large amount of experimental work has been carried out based on the high-pressure polymerization of C_60_, and it has been established that many polymerized phases can be produced under various pressure and temperature conditions, including 1D orthorhombic phase [197], 2D tetragonal [198] and rhombohedral phases [199] and 3D networks [38,109].

**Figure 9.**
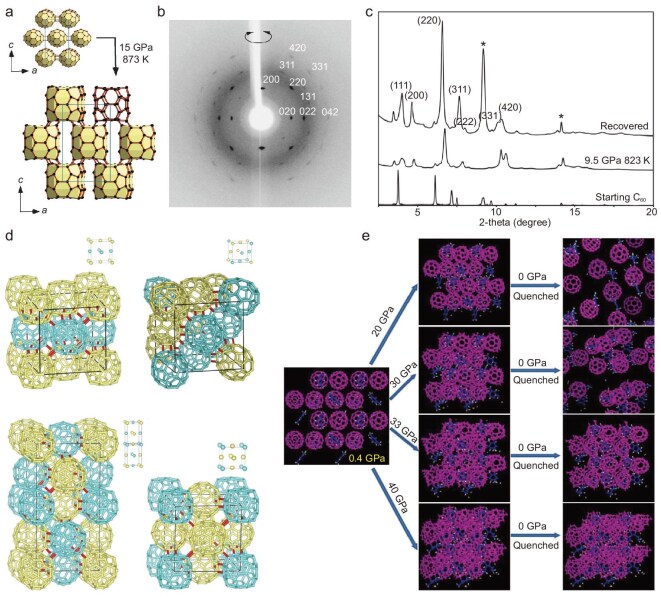
Fullerene-derived 3D carbons obtained by high-pressure treatment. (a) Structural transition of the 3D orthorhombic C_60_ polymer from 2D polymer. Adapted with permission from ref. [38]. Copyright 2006 American Physical Society. (b) XRD pattern of the 3D fcc C_60_ polymer crystal around the *a* axis. Adapted with permission from ref. [108]. Copyright 2008 American Chemical Society. (c) XRD pattern of an fcc polymeric C_60_ phase obtained at 9.5 GPa and 550°C (*λ* = 0.53396 Å). Adapted with permission from ref. [109]. Copyright 2017 John Wiley and Sons. (d) Ordered C_60_ binary-alloy-type structures including AuCuI-, CuPt-, ‘A_2_B_2_’- and Au_3_Cu-type used to describe the 3D fcc polymer obtained at 9.5 GPa and 550°C. Adapted with permission from ref. [204]. Copyright 2019 Elsevier. (e) Simulated structures and formation of ordered amorphous carbon clusters by compressing C_60_/*m*-xylene solvated crystals under different pressures. Adapted with permission from ref. [207]. Copyright 2012 American Association for the Advancement of Science.

3D C_60_ polymers, which can be obtained by applying a pressure higher than 8 GPa at high temperature, have been extensively investigated experimentally and theoretically [37,200–202]. 3D polymers can be roughly considered as 2D polymer sheets connected along the stacking directions, but in most cases 3D polymer samples synthesized under high pressure contain at least two phases, making the structural characterizations highly challenging. In 2006, Yamanaka *et al.* prepared a 3D polymer crystal by applying a pressure of 15 GPa to 2D tetragonal C_60_ polymer crystals at 600°C (Fig. 9a) [38]. Single crystal XRD suggests that it is a body-centered orthorhombic structure containing both *sp*^2^ and *sp*^3^ carbon atoms, in which the spherical C_60_ molecules deform into a cuboidal shape under high pressure, and each C_60_ is connected to eight adjacent molecules via [3 + 3] cycloaddition reactions. The resulting 3D polymer is electrically conductive with a conductivity of ∼10^−1^–10^−2^ S/cm at room temperature. Notably, the orientation and arrangement of C_60_ molecules in the 2D polymer remain unchanged after further high-pressure polymerization, even with the breaking and formation of intermolecular C-C bonds. Therefore, the *Immm* symmetry is maintained during the 2D–3D transition. Later in 2008, the same group compressed C_60_ single crystals to 15 GPa at 550°C with the addition of potassium azide (KN_3_) and obtained a new 3D polymer crystal with fcc symmetry [108]. Structural analysis based on XRD (Fig. 9b) results accompanied by *ab initio* MD calculations indicates that it is a rhombohedral phase crystal with the *R*-3 space group, in which each C_60_ unit is covalently connected to 12 adjacent units (6 units are bonded by [3 + 3] cycloaddition between adjacent 5-membered rings in the plane perpendicular to the 3-fold axis and another 6 units are bonded by 56/65 [2 + 2] cycloaddition from the bottom and top of the plane). Therefore, the obtained 3D fcc polymer is very hard, with Vickers hardness comparable to that of cubic boron nitride. Laranjeira *et al.* reported another 3D C_60_ polymer with fcc structure synthesized by compressing C_60_ powders at 550°C and 9.5 GPa [109]. Synchrotron radiation XRD (Fig. 9c) and calculations show that whether adjacent C_60_ molecules are bonded depends on their relative orientation, and such carbon crystals cannot be described by a defined crystal structure. The same group carried out further DFT calculations [203,204] and proposed a frustrated polymerized C_60_ structure with the long-range ordered fcc symmetry, in which the C_60_ molecules are bonded through 56/56 [2 + 2] cycloaddition, possibly between differently oriented neighboring molecules. Based on the 56/56 [2 + 2] cycloaddition intermolecular bonds, several polymerized structures have been constructed (Fig. 9d) and their stability and bulk moduli investigated. C_60_ polymers have been shown to have a range of interesting properties, including the semiconducting behavior of 1D/2D polymers, as well as the electroconductivity and extremely high hardness of 3D C_60_ polymers [37,38,205,206].

On the other hand, C_60_ molecules readily collapse under high pressures, and multiple covalent bonds can be formed between the deformed cages. Controlling the arrangement and polymerization of C_60_ under high pressures by introducing guest molecules into the C_60_ lattice is an effective strategy to synthesize 3D carbon-based crystals. In 2012, Wang *et al.* reported the preparation of a kind of long-range ordered carbon crystal called ordered amorphous carbon clusters (OACC) by compressing solvated C_60_ molecules [207]. The synthesis process is shown in Fig. 9e. Detailed XRD, Raman spectroscopy and MD simulation studies show that the position and structure of *m*-xylene molecules within the solvated crystals remain unchanged under high pressures, but the C_60_ cages gradually break down and form a periodic 3D structure. The OACC possesses superhard mechanical properties and remains stable after decompression. After that, a variety of solvated crystals composed of various fullerenes and solvent molecules were constructed, and the polymerization and phase transition processes at high pressures investigated in detail, illustrating the roles of building blocks and their boundary interactions in constructing novel carbon-based crystals [208–211]. Among solvent molecules, those with six-membered rings, such as *m*-xylene and 1,2,4-trimethylbenzene, tend to stabilize the highly compressed or collapsed fullerene clusters under high pressures, promoting the formation of long-range ordered structures. On the other hand, solvent molecules containing five-membered rings, such as ferrocene, usually transform into amorphous components together with fullerene cages, resulting in the formation of disordered structures under high pressures.

C_70_ molecule has an ellipsoidal shape with *D*_5h_ symmetry, which can form hexagonal close-packed molecular crystal at ambient conditions [212,213]. Soldatov *et al.* investigated the compressibility of C_70_ by means of direct piston and cylinder measurements at temperatures ranging from 150 to 365 K and pressure up to 1 GPa [214]. The phase transition from a simple rhombohedral phase to a fcc phase was found at 867 K and 0.9 GPa [213]. In 1998, Blank *et al.* investigated the phase transition of C_70_ at pressures up to 12.5 GPa and temperatures of 300 to 1770 K [215], and found a new superhard 3D-polymerized tetragonal phase at pressures above 9.5 GPa and a superhard disordered state at 12.5 GPa with a hardness close to that of (100)-face diamond. In 2000, four crystalline structures formed by dimerization and polymerization of C_70_ were identified by TEM of C_70_ treated at 9.5 GPa and 850 K [216]. All the phases are triclinic and exhibit distorted fcc lattices with a doubled *c*_0_-parameter. Soldatov *et al.* synthesized a C_70_ polymer by treating hexagonal close-packed C_70_ single crystals at 2 GPa and 300°C, confirming the predictions on the modeling of C_70_ polymers. Single crystal XRD shows that the C_70_ molecules are covalently connected into polymeric zigzag chains along the *c* axis of the parent structure. Solid-state nuclear magnetic resonance and Raman spectroscopy studies demonstrate the existence of covalent bonds between C_70_ cages [217].

We can see that the existing carbon structures can be compressed into new carbon allotropes such as M-, V-carbon, and 3D fullerene polymers by high-pressure treatment, although many structures formed under compression are unstable at ambient conditions. In addition, the limited sample size in the tiny anvil may hamper detailed characterizations of the sample obtained. Selecting suitable carbon precursors and regulating their stacking/orientation, combined with the control of temperature, pressure, shear and doping, may promote the experimental preparation of more 3D carbon crystals.

### Charge-injection-induced structure phase transition of carbons

Irradiation from an electron beam [218] or light [219], plasma treatment [220], alkali/alkaline-earth metal doping [221–227] and other means can also lead to polymerization of fullerenes, providing a method for the preparation of new 3D carbon crystals. In the 1990s, in the study of *A*_3_C_60_ (*A* = K, Rb, Cs) superconductors, it was found that C_60_ molecules would connect with each other when doped with a stoichiometric proportion of alkali metals (*A*_1_C_60_) [222,228]. Alkali metal doping has been considered to be driven by the electron transfer from the alkali metal with low work function to the molecular orbitals of fullerenes. The electrons in the triple degenerate *t*_1u_ orbitals on C_60_ can react with the empty orbitals of neighboring molecules to form covalent bonds, contributing to the formation of polymers by stepwise [2 + 2] cycloaddition reactions [229]. By this route, numerous metal-doped C_60_ polymers have been successfully prepared and their properties investigated, including 1D KC_60_ [221], RbC_60_ [222], CsC_60_ [223], Na_2_RbC_60_ [224] polymer chains, 2D Li_4_C_60_ [225], Na_4_C_60_ [226], Mg_4_C_60_ [227] polymer sheets and so on. The type and amount of doped metal significantly affect the bonding, polymer structure and properties. For example, Na_4_C_60_ is an intermolecular all-single C-C bonds connected polymer, while single C-C bonds and four-membered carbon rings both exist in Li_4_C_60_. In KC_60_, C_60_ is linearly connected by four-membered carbon rings, forming 1D polymerized chains while Li_4_C_60_ is a 2D polymerized structure. Among them, Li_4_C_60_ [230] and Mg_2_C_60_ [231] are good ionic conductors, while nonpolymeric *A*_3_C_60_ (*A* = K, Rb, Cs) and Ca_5_C_60_ exhibit superconductivity [228].

**Figure 10.**
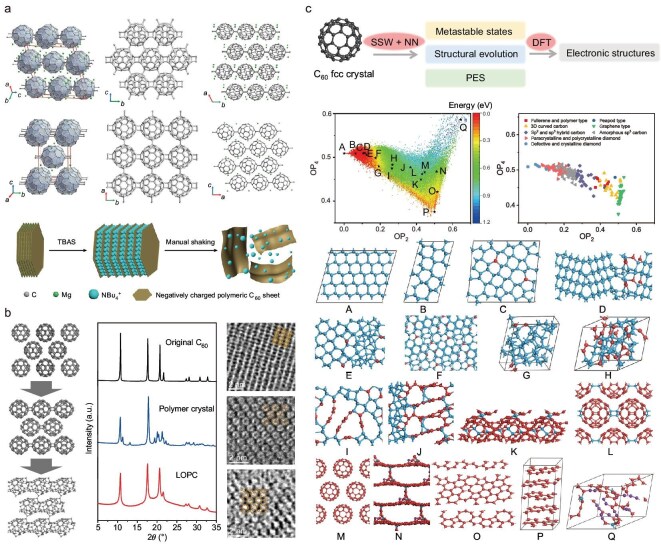
Charge-injection-induced structural transition of C_60_. (a) Crystal structures of Mg_4_C_60_ and Mg_2_C_60_ polymers determined by single-crystal XRD and a schematic of organic cation slicing exfoliation. Adapted with permission from ref. [40]. Copyright 2022 Springer Nature. (b) Charge-injection-induced structural transformation of C_60_ molecular crystal to 1D orthorhombic polymer and 3D fcc LOPC crystal, and the corresponding XRD and high-resolution TEM images. Adapted with permission from ref. [43]. Copyright 2023 Springer Nature. (c) 2D potential energy surface mapping and typical structures during structural evolution starting from C_60_ molecular crystal. Adapted with permission from ref. [233]. Copyright 2022 John Wiley and Sons.

Typically, only polycrystalline powder samples of fullerene polymers can be obtained, restricting the systematic study of the properties [226,227,232]. In 2018, Tanaka *et al.* synthesized single crystals of Mg_2_C_60_ polymer [39] via chemical vapor transport and provided more precise structural information of the 2D polymer network, e.g. detailed bond distances and carbon coordination surrounding the intercalated Mg atoms, through single crystal X-ray structural analysis. In 2022, Hou *et al.* altered the ratio of raw materials and achieved the preparation of 3D monoclinic Mg_4_C_60_ and Mg_2_C_60_ polymer crystals [40]. By characterizations, it was found that both polymer crystals are composed of tightly stacked 2D C_60_ polymer sheets connected by intercalated Mg ions, in which the Mg-C bonds exist in an intermediate state between covalent and ionic bonds. Figure 10a shows the crystal structure of these two samples. By organic cation slicing, the Mg_4_C_60_ polymer crystal was exfoliated into negatively charged, quasi-hexagonal polymer monolayers and dispersed in N-methylpyrrolidone (NMP) solution. Meirzadeh *et al.* also synthesized Mg_4_C_60_ crystals using the same synthesis route and obtained few-layer C_60_ polymer sheets by acid pickling and mechanical exfoliation, based on which the heat transport behavior was studied [41]. Preliminary property testing shows that the 2D C_60_ polymer owns enhanced thermal and electric conductivity compared with C_60_ molecular crystal because of in-plane covalent bonding.

The role of charge injection for modulating the structure of carbons has been further highlighted by our group. In 2021, Pan *et al.* reported the transformation of graphite from 3R to 2H phase by charge injection from lithium nitride (*α*-Li_3_N) [42]. DFT simulations and *in*-*situ* Raman spectroscopy show that *α*-Li_3_N transfers electrons to graphite, enhancing the interlayer repulsion at high temperature, thus promoting the interlayer slipping of graphite from 3R to a more stable 2H phase, as verified by *in*-*situ* XRD. Using the same idea, Pan *et al.* treated the mixture of *α*-Li_3_N and C_60_ powders under vacuum at temperatures of 440–600°C, successfully achieving the transformation of fcc C_60_ molecular crystals to 1D linear polymer crystals and 3D LOPC crystals in hours (Fig. 10b) [43]. In this way the yield is much improved, leading to the gram scale production of 3D carbons from C_60_ for the first time. Systematic characterizations indicate that the LOPC crystal is a new 3D carbon crystal formed by covalently bonded distorted C_60_ molecules, and is porous but retains the long-range ordered structure of C_60_ in molecular crystals. Theoretical simulations suggest that the electric dipole moments generated by charge injection into C_60_ molecules can propagate along neighboring molecules, reducing the energy barrier of additional reaction between C_60_ molecules. Through machine learning and neural network structure search, Ni *et al.* further demonstrated that LOPC crystals represent a large class of metastable crystal structures in the structural evolution from C_60_ molecular crystals to graphite-like or diamond-like carbon crystals (Fig. 10c), and the number of specific types of structures may be very large, from tens of thousands to a million [233].

Based on the crystals composed of metal and fullerene (mostly C_60_) cages, the recent advancements reviewed above have clearly shown that new 3D carbon crystals can be obtained after removing the metals, which are generally considered to stabilize the connection between fullerene molecules. Combined with the calculations which show a great number of metastable phases derived from C_60_, we can reasonably speculate that there is much room for the preparation of new 3D carbon crystals, e.g. by adjusting the experimental parameters of charge injection, including temperature, reaction atmosphere and pressure, doping amount and dimension. In addition, the examples above may remind us to correlate the charge-injection-induced structure change with the carbons used in the anodes of batteries, since the charge injection can obviously be better controlled in electrochemistry. In fact, the polymerization phase transition of C_60_ [234] and stacking phase transition of graphite [235,236] have been observed in the *in-situ* studies of carbon anodes for Li-ion batteries.

## SUMMARY AND PROSPECTS

So far, the most familiar 3D carbon crystals are soft/electrically conducting graphite and hard/insulating diamond, both of which have found numerous applications in social economy and human life. The novel 3D carbon crystals may demonstrate more superior properties by tuning the symmetry, controlling the connection between carbon units and inducing curvatures or metals in the crystal. Depending on these parameters, they can be semiconducting, superconducting, super hard, porous or colorful in sunshine. Such interesting properties may have great potential for applications in semiconducting devices, energy storage, species separation and so on. Although a great variety of new 3D carbon crystals have been proposed based on theoretical calculations, most of them are still to be experimentally synthesized. Therefore, the experimental preparation of novel 3D carbon crystals is a long-term goal in carbon nanoscience and intensively pursued by materials scientists.

From the perspective of the controllable synthesis of 3D carbon crystals, this review has summarized the research progress of utilizing existing carbons to construct 3D carbon crystals. Big challenges remain. Firstly, template-assisted preparation seems a useful technique for large-scale preparation but it is very challenging to verify the local atomic arrangement and bonding state. A promising research idea is to integrate template carbonization with HPHT processing, which may induce atomic rearrangement, local structures repair and further structural evolution, facilitating the obtaining of new *sp*^3^ or *sp*^2^-*sp*^3^ mixed hybridization porous carbons with better crystallinity and short-range order. Secondly, although organic synthesis can provide the control of structure and reaction at the atomic scale, the actual experimental research still remains in the design of molecular fragments with structural information of the target 3D carbon crystals. The next step could be to find appropriate templates, reaction systems or catalysts to control the precise reaction and 3D assembly of these nanoscale building blocks. Thirdly, although high-pressure processing of existing carbon materials has demonstrated its power for the synthesis of new carbon allotropes, more strategies need to be developed to stabilize the carbon crystals generated under high pressures. Using solvation, intercalation and surface modification to control the orderly arrangement and close packing of precursor molecules, combined with high temperature, shear and doping, is expected to achieve more experimental advancements with regard to the synthesis of new carbon crystals, which are still theoretical.

Recent work has highlighted the role of charge injection in the phase transition of fullerenes or graphite, providing a readily scalable strategy for the precise regulation of carbon structures at the atomic level. In particular, charge-injection-assisted synthesis can be carried out at atmospheric pressure and at reasonably acceptable temperatures, beneficial for mass production. To better tailor the structures of the 3D carbon crystals obtained, the bonding mechanism between fullerenes under atmospheric pressure is key. Combined with regulation of temperature, pressure and reactions, we believe there is potential to realize the preparation of more interesting 3D carbons based on the carbon nanostructures we already have, as a result of which, research into carbon materials science would also be greatly advanced.
